# Cu^3+^ Ion Evaluation and O^2−^ Vacancy Identification in CuO Nanofibers by XPS

**DOI:** 10.3390/ma19132689

**Published:** 2026-06-23

**Authors:** Manuel Piñon-Espitia, Saul Verdugo-Miranda, Rafael Verdugo-Miranda, Jose Duarte-Moller, M. T. Ochoa-Lara

**Affiliations:** 1 Department of Physics and Engineering, Universidad de Sonora, South Regional Campus, Lázaro Cárdenas del Río 100, Francisco Villa, Navojoa 85880, Sonora, Mexico; saul.verdugo@unison.mx (S.V.-M.); rafael.miranda@unison.mx (R.V.-M.); 2 Centro de Investigación en Materiales Avanzados S. C., Chihuahua 31136, Chih, Mexico; martha.ochoa@cimav.edu.mx

**Keywords:** nanofibers, electrospinning, XPS, CuO, Cu^3+^

## Abstract

Cu^3+^-like species and oxygen vacancies (Vₒ) in electrospun CuO nanofibers were identified by X-ray photoelectron spectroscopy (XPS) via Cu 2*p*^3/2^ and O 1*s* core-level spectra. Nanobeam electron diffraction (NBD) revealed a Cu^3+^-related superlattice. The geometrical topofactor method corroborated the chemical composition of samples thermally treated at 600 °C (CuO600) and 700 °C (CuO700). Bulk CuO served as a comparison. XPS peak fitting of the Cu 2*p* and O 1*s* regions used an SVSC-type background and a two-parameter Tougaard function. X-ray diffraction (XRD) confirmed the presence of tenorite and cuprite phases. Crystallite size was estimated using the Rietveld method; values ranged from 20.59 ± 0.06 nm to 31.06 ± 0.06 nm. High-resolution transmission electron microscopy (HR-TEM) produced sizes of 14.98 ± 0.34 nm and 36.10 ± 0.94 nm, highlighting the distinction between diffraction domains and physical particle dimensions. Cu^3+^-like species and oxygen vacancies modulate the nanofibers’ electronic structure, which is relevant to electronic applications.

## 1. Introduction

Copper oxides, Cu_x_O (x = +1, +2, +3) [1], are advanced materials with notable optical, electrical, thermal, and magnetic properties, extensively studied for their applications. This research specifically focuses on the role of Cu^3+^-like species and oxygen vacancies (VO) in one-dimensional CuO nanostructures, examining how these defects influence structure–property relationships, particularly in nanofiber forms of cuprous oxide (Cu_2_O, cuprite) and cupric oxide (CuO, tenorite) [2].

CuO, owing to its multivalent nature and high density of oxygen vacancies (Vₒ), exhibits properties of great interest for applications in sensors, photodetectors, photocatalysis, energy conversion, and optoelectronic devices [3,4,5,6,7,8,9]. Oxygen vacancies—dominant defects in CuO nanostructures—modify the electronic structure, enhance charge carrier mobility and surface reactivity, and may even induce morphological changes [7,9,10,11]. The electrophysical properties of CuO- and Cu_2_O-based nanostructures are strongly governed by defect concentration, crystallite size, and the coexistence of mixed copper valence states [3,6,11]. In this context, Cu^3+^-like species promote charge-transfer processes, alter the defect landscape, and modulate electrical conductivity, dielectric response, and the band gap, which is sensitive to both quantum confinement and stoichiometry [5,10]. In particular, direct band gap values of 1.49–1.79 eV have been reported for nanostructured CuO, exceeding those of single-crystal CuO (1.2–1.4 eV) and commonly attributed to quantum confinement effects and oxygen vacancies [6,10].

As noted by Sarkar et al. [1] and Collins et al. [12], the presence of Cu^3+^-like species is correlated with excess hole concentration and contributes to the semiconducting character of CuO. Their studies, combining thermal treatments with X-ray photoelectron spectroscopy (XPS) and selected-area electron diffraction (SAED), revealed a small but measurable concentration of Cu^3+^-like species that increases with temperature, thereby enhancing both the dielectric response and semiconducting behavior. Similar findings were reported by Raj et al. [2], who associated the improved conductivity of nanostructured CuO with an increased Cu^3+^ content and a secondary contribution from oxygen vacancies (Vₒ).

XPS helps identify copper oxidation states; the Cu 2*p* region gives key clues about Cu^3+^-related signals. However, interpreting these spectra can be difficult due to background noise, spectral shape, and multiplet patterns. These factors make CuO spectra more complex than other transition-metal oxides [13,14,15,16,17,18].

The objective of this work is to clarify how Cu^3+^-like species, in connection with oxygen vacancies, affect structure–property relationships in one-dimensional CuO nanofibers. Using XPS, HRTEM, Rietveld refinement, and NBD, we systematically analyze the impact of thermal treatment on Cu^3+^ concentration and its interplay with defect features. Electrospun CuO nanofibers treated at 600 °C and 700 °C and a bulk CuO reference are compared to provide comprehensive insight into defect-driven behaviors relevant to device applications.

## 2. Experimental Procedure

Polymeric precursor fibers were produced by electrospinning. An 8 wt% poly(vinyl alcohol) (PVA; Sigma-Aldrich, St. Louis, MO, USA, Mw ≈ 130,000, high purity) solution was prepared by dissolving 8 g of PVA in 92 mL of triple-distilled water (resistivity 1.1 MΩ·cm; J. T. Baker, Phillipsburg, NJ, USA) with magnetic stirring for 24 h at room temperature.

A copper precursor solution was prepared by dissolving 1 g of copper (II) acetate (Sigma-Aldrich, St. Louis, MO, USA, 99%) in deionized water, then stirring for 4 h at 50 °C. Subsequently, 20 g of the PVA solution was added to the copper precursor solution, and the mixture was stirred at 600 rpm for 24 h until a homogeneous and transparent solution was obtained.

A volume of 10 mL of the final solution was loaded into a syringe mounted on a syringe pump as part of the electrospinning setup. An 8 kV voltage was applied between the needle and an aluminum foil-covered collector, separated by 20 cm.

Thermal analysis (TGA/DSC) guided the choice of calcination temperatures. Electrospun fibers were heated in air at 10 °C·min^−1^ to 600 °C and 700 °C. This produced CuO600 and CuO700 samples. Heating converted precursor fibers into CuO nanofibers and removed organic residues (PVA and acetate compounds).

## 3. Characterization

### 3.1. Structural Characterization by XRD

A high-resolution X-ray diffractometer (PANalytical X’Pert PRO, Panalytical B.V., Almelo, The Netherlands) with an X’Celerator detector (Panalytical B.V., Almelo, The Netherlands) analyzed the crystalline structure of CuO nanofibers. Diffraction data used Cu Kα radiation (λ = 1.5418 Å), a 2θ range of 30–90°, a step size of 0.01°, and a counting time of 0.01 s per step. This setup enabled the detection of minor Cu_2_O reflections and the identification of small phases.

Rietveld refinement for structural analysis was performed using the FullProf Suite software 2026 [19,20]. XRD data were formatted and refined with the EDT/PCR module using a non-linear least-squares fitting. A TCH pseudo-Voigt profile function modeled peak shapes and estimated average crystallite size.

For the main CuO phase, a monoclinic structural model corresponding to the space group *C2/c* was used, incorporating reported lattice parameters and atomic positions: Cu at the 4c (1/4, 1/4, 0) site and O at the 4e (0, y, 1/4) site, with y ≈ 0.418 [21]. The background was modeled using a polynomial function, and the refinement included corrections for axial divergence, as well as the use of an instrumental resolution file (xray-res.if) to improve the fit quality.

This method extracted microstructural details, like crystallite size. For CuO600, the refinement included cuprite (Cu_2_O) as a secondary phase with its structural parameters. The identified phases matched standard PDF cards 80-1268 (CuO) and 75-1531 (Cu_2_O).

Finally, the refined results were exported and processed using OriginPro software 2024 [22] for statistical analysis and graphical representation.

### 3.2. Scanning Electron Microscopy (SEM)

Samples were prepared by cutting approximately 1 cm^2^ sections from the aluminum foil collector used during electrospinning. Scanning electron microscopy (SEM) micrographs were acquired using a Hitachi SU3500 microscope (Hitachi High-Tech Corporation, Tokyo, Japan).

Fiber diameters (SEM) and nanofiber diameters (TEM) were measured in ImageJ software 2017. Multiple regions were selected randomly for statistical accuracy. Transverse measurements were taken at various points along each fiber.

The resulting data were exported to OriginPro software 2024 [22] for statistical analysis, including histogram construction and log-normal distribution fitting. This approach enabled an accurate characterization of the size dispersion and statistical distribution of the fiber diameters.

### 3.3. High-Resolution Transmission Electron Microscopy (HRTEM)

Specimens were dispersed in isopropanol (Sigma-Aldrich, St. Louis, MO, USA, 99.8%) for 1 h. A drop of suspension was placed on 3 mm Ni grids and allowed to dry. HRTEM images were taken with a JEOL JEM-2200FS microscope (JEOL Ltd., Akishima, Japan) and processed using Digital Micrograph software 2023 [23].

### 3.4. X-Ray Photoelectron Spectroscopy (XPS)

XPS measurements used a Thermo Scientific ESCALAB Xi instrument (Thermo Fisher Scientific, Waltham, MA, USA) with a monochromatic Al Kα source (*hν* = 1486.7 eV). Acquisition parameters: energy step of 0.1 eV, dwell time of 200 ms, 40 scans, and takeoff angle of 90°. CuO nanofibers were mounted on conductive graphite tape. Samples were loaded into the vacuum chamber (10^−6^ Torr), then moved to the analysis chamber for measurements at ~10^−6^ Torr. The monochromator was set at 45° to the collection axis.

### 3.5. Data Analysis

Peak fitting of the Cu 2*p* and O 1*s* regions was performed using A Analyzer^®^ software 2026, employing a Shirley/SVSC-type background, a two-parameter Tougaard function, and slope correction. The chemical composition was determined using the geometrical top-down approach based on a spherical model. Calculations were carried out using the Cumpson core–shell model [24,25].

## 4. Results and Discussion

### 4.1. XRD Analysis

**Figure 1:**
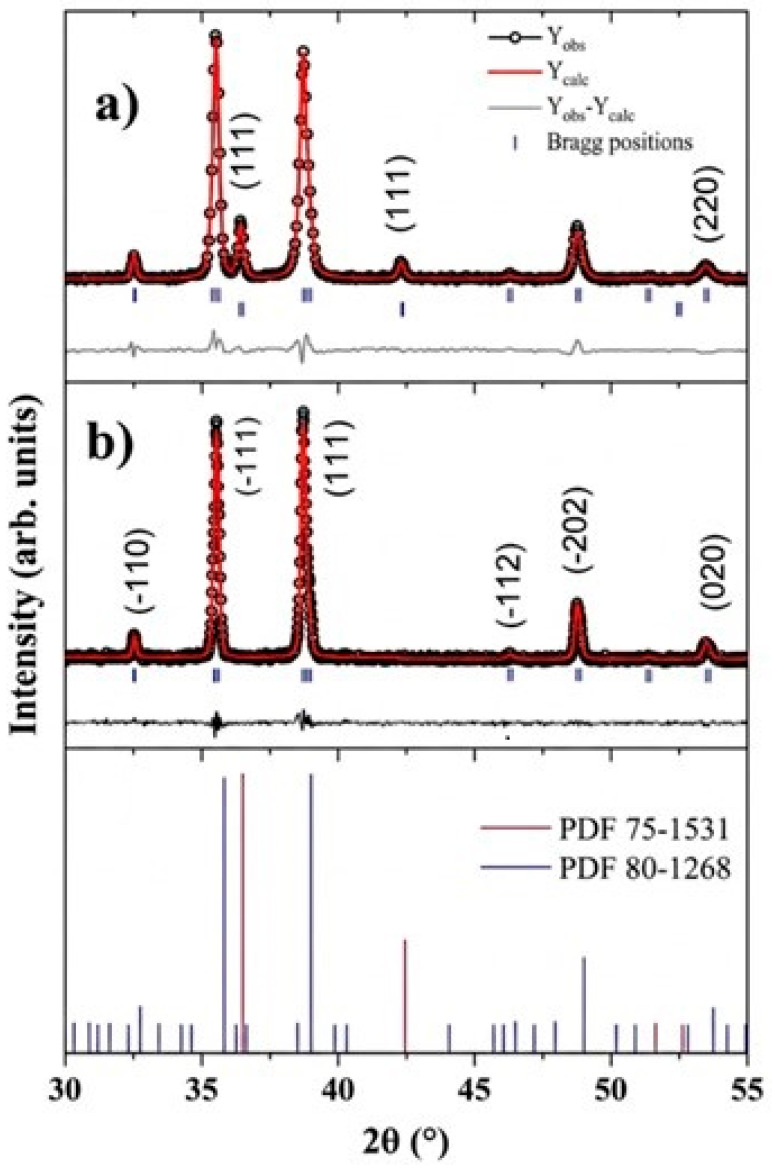
XRD patterns and Rietveld refinements for (**a**) CuO600 and (**b**) CuO700, performed using the FullProf Suite [26,27].

Structural characterization of the CuO600 and CuO700 samples was performed by Rietveld refinement of the XRD patterns using FullProf software. The refined diffraction profiles are shown in Figure 1a, where the calculated patterns (red line) agree well with the experimental data, confirming the reliability of the refinement procedure.

The CuO600 sample exhibits a mixed-phase composition, consisting predominantly of monoclinic CuO (tenorite, PDF 80-1268) with a minor contribution from Cu_2_O (cuprite, PDF 75-1531), as evidenced by the presence of additional reflections (Figure 1b). In contrast, the CuO700 sample shows a single-phase CuO structure, indicating that higher calcination temperature promotes phase stabilization and the complete transformation of residual Cu_2_O into CuO.

Subtle variations in peak positions and relative intensities are observed between the two samples, suggesting slight changes in lattice parameters and crystallite size associated with the thermal treatment. These results indicate that increasing the calcination temperature enhances structural ordering and phase purity in the CuO nanofibers.

The refinement results obtained using FullProf are summarized in Table 1.

**Figure 2:**
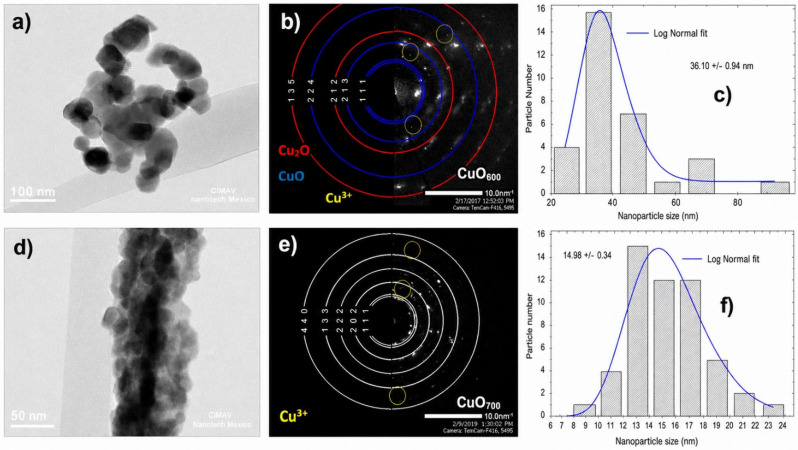
CuO600 nanofibers: (**a**) TEM micrograph showing the morphology, (**b**) corresponding nano-beam electron diffraction (NBD) pattern, and (**c**) particle/nanofiber size distribution histogram. CuO700 nanofibers: (**d**) TEM micrograph showing the morphology, (**e**) corresponding NBD pattern, and (**f**) particle/nanofiber size distribution histogram.

The average crystallite sizes estimated by XRD for CuO600 and CuO700 were 31.06 ± 0.06 nm and 20.59 ± 0.06 nm, respectively. In comparison, the particle size distributions obtained from TEM (Figure 2c,f) follow a Log-normal behavior, with average values of 36.10 nm and 14.98 nm. These differences reflect the effect of thermal treatment and are consistent with the peak broadening observed in the XRD patterns (Figure 1).

The discrepancy between XRD and TEM results arises from the intrinsic differences between both techniques. While XRD provides an average crystallite size based on diffraction domain coherence, TEM measures the physical particle size. As widely reported in the literature [28,29,30,31,32,33,34,35,36,37], such variations are commonly associated with limitations in modeling diffraction profiles using a single set of microstructural parameters, which may increase fitting uncertainty and lead to non-coincident values.

Additional insight is provided by Williamson–Hall (W–H) analysis [21], which suggests that CuO formation proceeds from Cu_2_O through oxygen diffusion processes. In this context, the coexistence of phases is not primarily associated with lattice strain but rather with crystallochemical stability. The reduction in crystallite size observed for CuO700 is therefore attributed to kinetic effects, atomic diffusion, and reduced thermodynamic stability during thermal treatment [21,38].

The correlation between XRD and XPS results for CuO600 and CuO700 highlights the relevance of microstructural changes associated with crystallite size reduction. Although the values obtained by XRD and TEM are not strictly consistent, both techniques indicate characteristic dimensions below 50 nm, which favor modifications in the chemical nature of the Cu–O bond.

According to previous CTM4XAS (XPS) analyses, the Slater integral indicates an electronic contribution of d^9^ = 76% and d^10^L = 24%, confirming a significant mixed ionic–covalent character of the oxide [21]. This electronic structure directly influences the material’s electrophysical properties [32].

### 4.2. SEM, HR-TEM, and NBD Analysis

**Figure 3:**
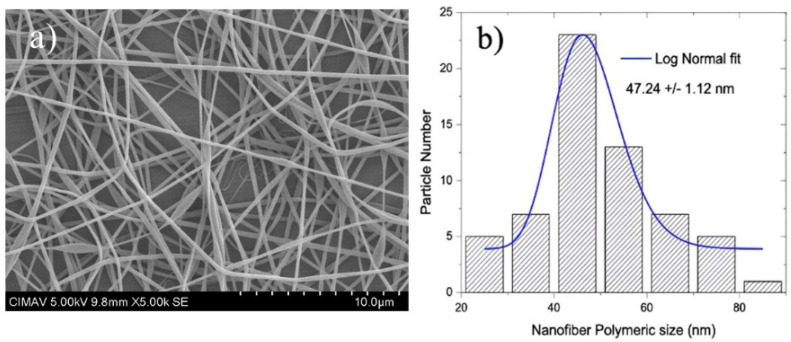
Electrospun precursor fibers. (**a**) As-spun composite/polymeric nanofibers consisting of PVA loaded with copper (II) acetate, and (**b**) nanofiber diameter distribution histogram.

The SEM micrograph in Figure 3a shows the as-electrospun polymeric precursor nanofibers, consisting of PVA containing copper (II) acetate, forming a randomly oriented fibrous network. Figure 3b presents the corresponding diameter distribution of the as-spun fibers.

Prior to calcination, the precursor fibers consisted of a polymer–metal salt composite. Upon thermal treatment at 600 °C and 700 °C (yielding CuO600 and CuO700, respectively), the organic matrix was removed and the acetate species decomposed, leading to the formation of CuO nanofibers.

The calcined products (CuO nanofibers) were examined by HRTEM and nano-beam electron diffraction (NBD) (Figure 2). The representative morphologies of CuO600 and CuO700 are shown in Figure 2a,d, respectively. The corresponding NBD patterns (Figure 2b,e) were indexed using CrysTBox 2026 [39], confirming the presence of the tenorite (CuO) phase in both samples, in agreement with the XRD results.

In Figure 2b,e, the reflections obtained by NBD for CuO600 and CuO700 suggest the presence of Cu^3+^-related features (highlighted by the yellow circles). In line with previous reports, these features have been described as superlattice-like contributions associated with additional ionic species. Such contributions are not resolved by XRD, likely because of their low concentration; they may instead reflect local charge density variations, defect-related signatures (e.g., vacancies), and/or minor phases present at trace levels [1,40].

### 4.3. XPS Analysis and Chemical Composition

**Figure 4:**
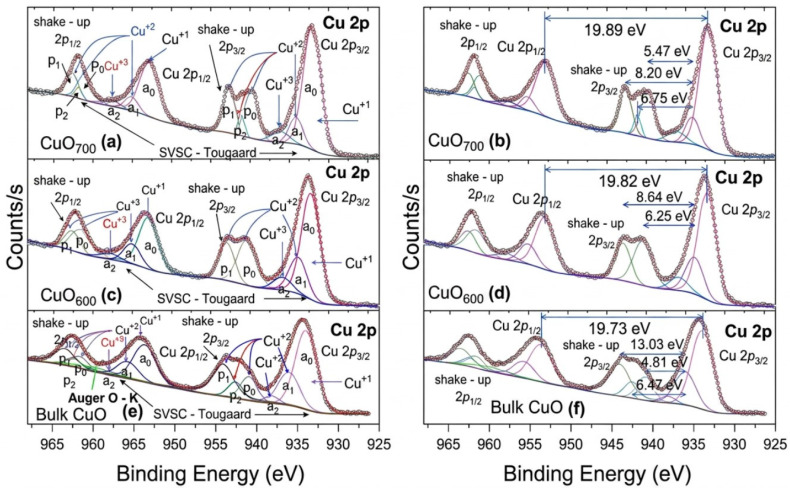
Cu 2p XPS spectra and spin–orbit splitting between the Cu 2*p*^3/2^ and Cu 2*p*^1/2^ components for CuO700 (**a**,**b**), CuO600 (**c**,**d**), and the bulk reference sample (**e**,**f**).

The XPS results for CuO600 (Figure 4c,d) and CuO700 (Figure 4a,b) indicate the presence of Cu^3+^-related contributions in the Cu 2*p* region (j = 3/2 and j = 1/2), evidenced by features at 936.81 eV and 937.10 eV, respectively. In contrast, these features were not observed in the bulk CuO reference (Figure 4e,f). An additional contribution associated with the Cu 2*p*^3/2^ shake-up satellite was detected in the CuO700 and reference spectra, but it was absent in the CuO600 spectrum. The origin of this satellite feature remains debated; it has been linked to chemisorption effects and/or an excess of copper species at the surface [1,41,42]. The absence of suppressed satellites in CuO600 suggests a reduced surface disorder rather than a direct compositional change. This alternative explanation invites further critical engagement with spectral data. Table 2 summarizes the peak areas obtained from the fitting of the Cu 2*p* and O 1*s* spectra. The table also lists the binding-energy assignments for the proposed chemical species in the Cu 2*p* region (Cu^1+^, Cu^2+^, and Cu^3+^) and the O 1*s* region (O^2−^) for CuO600, CuO700, and bulk CuO. Only minor variations were observed when using Gaussian–Lorentzian (GL) line shapes (see Table 3), and the fitted parameters remained consistent with previously reported values; a detailed discussion of the fitting theory is beyond the scope of this work [17].

**Figure 5:**
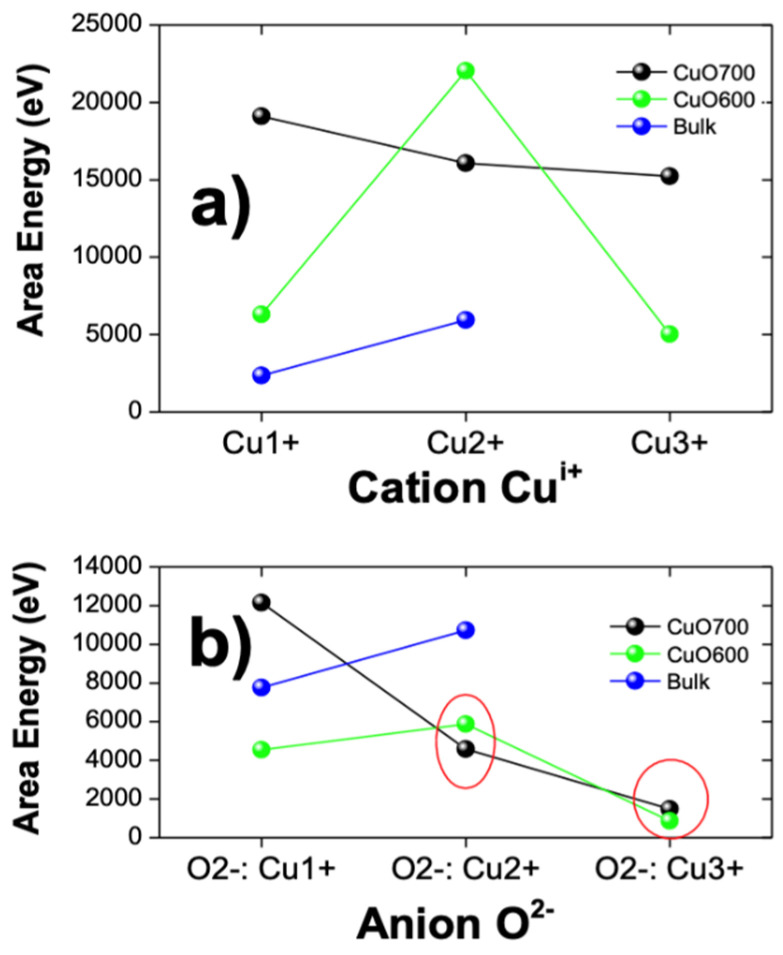
Schematic representation of the fitted peak areas derived from the Cu 2*p* and O 1*s* XPS spectra. (**a**) displays the calculated areas corresponding to the Cu^1+^, Cu^2+^, and Cu^3+^ cations in the Cu2*p* spectrum; (**b**) corresponds to the O^2−^ peaks.

In Figure 5, the fitted peak areas are compared across the proposed chemical species, showing the relationship between the peak area and the Cu oxidation states (Cu^1+^, Cu^2+^, and Cu^3+^) (Figure 4a). In Figure 5a for the Cu^1+^ component, CuO600 and the bulk reference exhibit similar areas (≈835.7 a.u.), whereas CuO700 shows a markedly higher value (≈19,000 a.u.). For the Cu^2+^ component, the CuO600 and CuO700 samples fall in the range of approximately 16,000–22,000 a.u., whereas the bulk sample shows a substantially lower area (≈5035 a.u.). Finally, the Cu^3+^-related component exhibits comparable areas in CuO600 and CuO700 (≈11,587.6 a.u.) and was not observed in the bulk reference. Overall, this indicates a trend toward an oxygen-rich but Cu^1+^-dominant chemical landscape, particularly in the CuO700 sample, where Cu^1+^ was significantly more pronounced.

In Figure 5b, the O^2−^ anion contribution is compared with the previously discussed cationic components through the ratios O^2−^:Cui (with *i* = 1^+^, 2^+^, 3^+^). The O^2−^:Cu^3+^ ratio yielded values of approximately 2000 a.u. for both CuO600 and CuO700. For O^2−^:Cu^1+^, the minimum value was observed for CuO700 (≈4000 a.u.), whereas the bulk reference and CuO600 showed higher values of approximately 8000 a.u. and 12,000 a.u., respectively. In addition, O^2−^:Cu^2+^ for CuO600 and CuO700 lies in the range of approximately 4000–6000 a.u., whereas the bulk sample reaches a maximum of approximately 10,000 a.u.

Cu 2*p* XPS spectra and the spin–orbit splitting between the Cu 2*p*^3/2^ and Cu 2*p*^1/2^ components for CuO600 (Figure 4c,d), CuO700 (Figure 4a,b), and bulk reference samples (Figure 4e,f), respectively.

The spin–orbit splitting between the Cu 2*p*^3/2^ and Cu 2*p*^1/2^ components was evaluated for CuO600 (Figure 4c,d), CuO700 (Figure 4a,b), and the bulk reference sample (Figure 4e,f). The Cu 2*p*^3/2^ and Cu 2*p*^1/2^ components are shown in Figure 4b,d,f. A slight decrease in the binding energy was observed, which may be associated with changes in the electronic structure of the Cu 2*p* levels (with degeneracy) [43]. The splitting of the main Cu 2*p* peaks was estimated from the measured positions of the Cu^2+^ components and the corresponding Cu 2*p*^3/2^ shake-up satellite features, yielding values in close agreement with those reported by Pauly, Tougaard, and Yubero [43].

In relation to the crystallochemical changes discussed in Section 4.1, the spectra shown in Figure 4 reveals the presence of multivalent copper states, consistent with the structural evolution described above. Notably, the CuO700 sample (panel a) and the bulk CuO reference (panel f) exhibit a higher intensity of shake-up features compared to CuO600 (panel c), indicating a greater density of hole states associated with charge-transfer processes.

These electrophysical variations suggest improved electrical conductivity and charge-carrier transport, in agreement with previous reports [11].

Table 3 lists the binding energies obtained from the fitting of the main Cu 2*p* peaks for CuO600, CuO700, and the bulk reference sample. The corresponding fitting errors were 3.72 × 10^−5^ eV, 2.57 × 10^−5^ eV, and 3.06 × 10^−5^ eV, respectively, confirming the high quality of the fitting procedure.

The fitting parameters associated with the SVSC Shirley-type and Tougaard backgrounds were consistent with previously reported values [1,2,43,44,45]. The Gaussian and Lorentzian broadening components exhibited values characteristic of nanostructured systems (Table 3). In contrast, the bulk reference sample showed no significant Lorentzian contribution, while the Gaussian broadening remained ≤ 3.0 eV, consistent with its larger structural scale.

Table 4 summarizes the background parameters used for the deconvolution of the Cu 2*p* spectra and their assignment to the proposed chemical species. Background modeling was performed using a combined SVSC Shirley-type and Tougaard approach, enabling reliable identification of the chemical components in the nanostructured samples. The extracted background energy parameters (Table 4) fall within the ranges reported in the literature [46,47,48,49,50]. Additionally, the degree of oxygen homogeneity was evaluated using the Shirley slope parameter [14].

**Figure 6:**
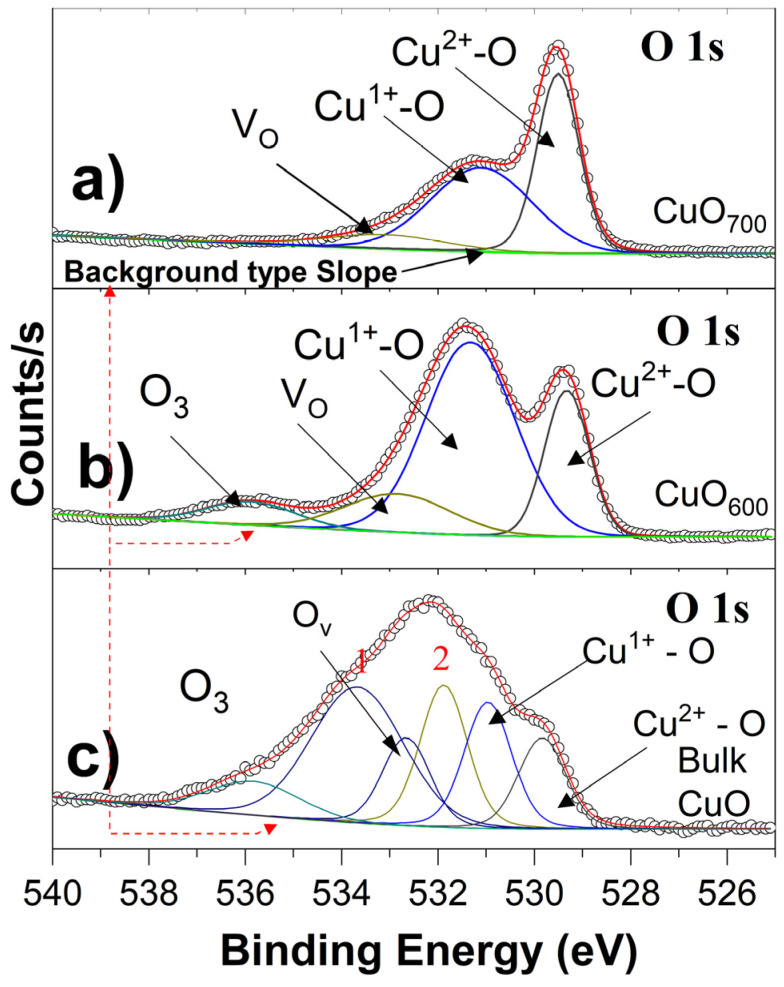
O 1*s* spectra for (**a**) CuO700, (**b**) CuO600, and (**c**) the bulk CuO sample.

Figure 6 shows the O 1*s* spectra of the samples and their relationship with the Cu 2*p* chemical components. The observed asymmetry is attributed to variations in oxygen coordination within the tenorite (CuO) structure [51]. Unlike previous studies that employed purely Gaussian functions [2,44,51], the O 1*s* envelopes were fitted with a combined Gaussian–Lorentzian (GL) line shape, yielding an optimized fit consistent with the observed spectral broadening [14,44].

In connection with the crystallochemical changes discussed in Section 4.1, the evolution of the Cu–O bonding environment is evident. The Cu^1+^–O component shows higher intensity in the CuO600 sample, reflecting the presence of a cuprite (Cu_2_O) phase, whereas the Cu^2+^–O contribution is more pronounced in CuO700, consistent with the formation of a more stable CuO phase. In addition, the commercial bulk CuO (panel c) shows a higher relative contribution of cuprite than CuO700.

According to previous studies [21], CuO600 exhibits a larger Cu–O interatomic distance (~2.35 Å) than CuO700 (~1.75 Å), which is associated with lower crystallinity. In contrast, increased crystallinity in CuO700 enhances oxygen coordination, thereby improving optoelectronic properties, such as electrical conductivity and band-gap behavior [10,21].

For CuO, the main lattice oxygen contribution (O^2−^) appears at 529.50 eV (CuO600), 529.30 eV (CuO700), and 529.76 ± 0.05 eV (bulk). Additional components at 531.15 eV (CuO600), 531.33 eV (CuO700), and 530.66 ± 0.05 eV (bulk) are assigned to higher-binding-energy oxygen species, commonly associated with oxygen-deficient regions (VO) [2,51].

Features at higher binding energies, such as 533.13 eV (CuO600) and 532.84 ± 0.01 eV (bulk), are attributed to surface oxygen species. In the bulk sample, an additional peak near ~536 eV is observed, which is assigned to adventitious carbon/CO_2_-related species (O_3_), likely originating from the carbon tape used during XPS measurements.

Additionally, a decrease in the O_3_ component (Figure 6b,c), located at 535.83 eV in CuO600 and 535.75 eV in the bulk sample, is observed. This component is typically associated with adsorbed molecular species or surface hydroxyl groups. Its reduction indicates a cleaner and less defective surface, consistent with thermally induced dihydroxylation processes commonly reported for copper oxides [10,11].

The stoichiometries calculated using the topofactor method were, for CuO600, Cu1.02O1.16, Cu2.22O2.12, Cu2.22O3.03; for CuO700, Cu1.02O1.15, Cu2.06O2.03, Cu2.05O3.19; and for the bulk sample, Cu0.97O1.02, Cu2.43O2.01, with an estimated error of ~4%. These values are supported by the geometrical core–shell model (cylindrical geometry) proposed in the literature [24,25,52,53].

Table 5 summarizes the chemical compositions derived from the primary XPS signals (Cu 2*p* and O 1*s*). The observed non-stoichiometric ratios may arise from excess oxygen, heat treatments conducted under uncontrolled atmospheres, and/or structural defects, including oxygen vacancies [44,54,55].

## 5. Conclusions

XRD analysis using the FullProf suite software revealed that CuO600 consists of a mixed-phase system composed of Cu_2_O (cuprite) and CuO (tenorite), with an average crystallite size of 31.06 ± 0.06 nm. In contrast, CuO700 exhibits single-phase CuO (tenorite) nanofibers with a reduced crystallite size of 20.59 ± 0.06 nm, indicating enhanced phase stability and structural ordering at higher calcination temperature.

NBD analysis of both CuO600 and CuO700 nanofibers revealed superlattice reflections associated with the presence of Cu^3+^-like species [1,40]. These findings are supported by XPS results, which confirm the coexistence of Cu^1+^, Cu^2+^, and Cu^3+^ oxidation states in the nanofiber samples, whereas the Cu^3+^ contribution is absent in the bulk CuO reference.

The Cu 2*p* spin–orbit splitting values (ΔE = E2*p*^1/2^ − E2*p*^3/2^) were determined to be 19.79 eV for CuO600, 19.89 eV for CuO700, and 19.92 eV for bulk CuO, consistent with previously reported values [1,2,16,43,44,56,57,58]. These variations suggest subtle changes in spin–orbit coupling associated with exchange interactions involving Cu 3*d* states [47].

The fitted XPS spectra indicate non-stoichiometry in the nanofibers, likely arising from calcination under non-controlled atmospheres, in agreement with compositional analysis [2,24,25]. The O 1*s* spectra further reveal that CuO600 exhibits a more pronounced oxygen-vacancy-related component (~531.33 eV), whereas CuO700 shows a reduced oxygen-vacancy concentration but a relatively higher contribution from Cu^3+^ states. The bulk reference sample lacks the Cu^3+^ feature near ~937 eV but shows a strong contribution in the oxygen-vacancy region.

Overall, the results demonstrate that both pure and mixed-phase CuO nanofibers undergo significant crystallochemical modifications, driven by their mixed ionic–covalent bonding character, as evidenced by combined XRD and XPS analyses. These modifications are strongly influenced by oxygen vacancies and multivalent copper states. The higher calcination temperature in CuO700 promotes increased crystallinity and reduced oxygen vacancy concentration compared to CuO600 and the bulk reference, while simultaneously favoring the stabilization of Cu^3+^ -like species.

## Figures and Tables

**Table 1:** Refined lattice parameters for CuO600 and CuO700 obtained by Rietveld analysis using the Thompson–Cox–Hastings pseudo-Voigt peak-shape function with axial-divergence asymmetry.

Nanostructure	A (Å)	b (Å)	c (Å)	V (Å^3^)	R_p_ (%)	R_wp_ (%)	R_exp_ (%)	χ^2^	Y
80-1268	4.6837	3.4226	5.1288	82.2830	-	-	-	-	-
75-1531	4.2678	4.2678	4.2678	77.31	-	-	-	-	-
CuO_700_	4.6858	3.4230	5.1298	82.2828	4.73	6.35	5.29	1.44	0.4184
CuO_600_	4.6860	3.4250	5.1343	81.297	12.8	18.2	17.70	1.05	0.41354
	4.2699	4.2699	4.2699	77.774					

**Table 2:** Peak areas obtained from fitting the Cu 2*p* and O 1*s* spectra, where A denotes the anion contribution and C the cation contribution.

Sample	TakeOff Angle	Cu^1+^ 2*p* (eV)	O 1*s* Cu^1+^ A (eV)	Cu^2+^ 2*p* (eV)	O 1*s* Cu^2+^ (eV)	Cu^3+^ 2*p* (eV)
CuO600	90	19,104.6	12,159	16,315.5	4590.52	4357.15
CuO700	90	6063.7	4543.56	22,029.7	5865.18	15,944.72
Bulk	90	5228	7766.1	5035.3	10,721	-

**Table 3:** Parameters used for fitting the Cu 2*p* region for each sample.

Sample	Date	Peak	2*p*^3/2^ BE (eV)	2*p*^1/2^ BE (eV)	Shake-Up 2*p*^3/2^ BE (eV)	Peak Width FWHM	
						Gaussian (eV)	Lorentzian (eV)
Bulk	Cu 2*p*	a_1_	933.72	953.58		3.78–3.30	0.085
		a_2_	935.35	955.8		2.79–3.41	0.085
		p_1_			941.59	3.22	0.085
		p_2_			944.11	2.86	0.085
		p_3_			943.72	1.00	0.085
CuO_600_	Cu 2*p*	a_1_	933.37	953.19		2.48	0.270
		a_2_	934.91	955.21		1.86	0.270
		a_3_	936.81	957.91		2.48	0.270
		p_1_			941.19	1.97	0.270
		p_2_			943.66	1.74	0.270
CuO_700_	Cu 2*p*	a_1_	933.19	953.09		2.48	0.270–0.514
		a_2_	935.17	955.28		1.86	0.270–0.059
		a_3_	937.1	957.67		2.48	0.270–0.514
		p_1_			940.64	1.97	0.27
		p_2_			943.37	1.74	0.27
		p_3_			941.91	1.00	0.27

**Table 4:** Parameters obtained from the SVSC (Shirley-type) background, Tougaard background, and slope term, derived from the optimized fitting of the Cu 2p and O 1*s* spectra.

Sample	Shirley Type SVSC (eV^−1^)	Shirley Type Tougaard (eV^−1^)	Shirley Type Slope (eV^−1^)
Bulk	0.028, 0.045	2000	0.0040
CuO600	0.035, 0.048, 0.035	2000	0.0025
CuO700	0.054, 0.068, 0.75	2000	0.0038

**Table 5:** Quantification of the primary XPS signals for the Cu 2*p* and O 1*s* peaks. Fitting peaks for the Chemical species Cu^1+^, Cu^2+^, Cu^3+^ and the variation with the calcination temperatures.

Sample	Ion		Peak BE (eV)	Peak BE (eV)	Cu*_i_*O	X_CuO_	X_C_	I_1_/I_2_	A	SF
CuO700	Cu^+1^	O^−2^	933.19	529.50	Cu^+1^-O^−2^	0.33–0.54		0.35–0.64	0.98	Cu_1.02_O_1.16_
	Cu^+2^	O^−2^	935.17	531.15	Cu^+2^-O^−2^	0.34–0.65		0.75–1.29	0.98	Cu_2.22_O_2.15_
CuO600	Cu^+1^	O^−2^	933.36	529.3	Cu^+1^-O^−2^	0.33–0.54		0.35–0.64	0.98	Cu_1.02_O_1.15_
	Cu^+2^	O^−2^	934.91	531.33	Cu^+2^-O^−2^	0.33–0.57		1.05–0.64	0.98	Cu_2.06_O_2.03_
Bulk	Cu^+1^	O^−2^	933.33	529.76	Cu^+1^-O^−2^	0.35–0.48		0.35–0.51	0.98	Cu_0.97_O_1.02_
	Cu^+2^	O^−2^	936.06	530.66	Cu^+2^-O^−2^	0.81–0.54		1.66–1.36	0.98	Cu_2.43_O_2.01_

## Data Availability

The original contributions presented in this study are included in the article. Further inquiries can be directed to the corresponding authors.
